# Australian Consumers’ Awareness and Acceptance of Insects as Food

**DOI:** 10.3390/insects9020044

**Published:** 2018-04-19

**Authors:** Kerry Wilkinson, Beverly Muhlhausler, Crystal Motley, Anna Crump, Heather Bray, Rachel Ankeny

**Affiliations:** 1 School of Agriculture, Food and Wine, The University of Adelaide, Waite Campus, PMB 1, Glen Osmond 5064, South Australia, Australia; beverly.muhlhausler@adelaide.edu.au (B.M.); crystalangelamotley@outlook.com (C.M.); anna.crump@adelaide.edu.au (A.C.); 2 School of Humanities, The University of Adelaide, Adelaide 5005, South Australia, Australia; heather.bray@adelaide.edu.au (H.B.); rachel.ankeny@adelaide.edu.au (R.A.)

**Keywords:** consumer acceptance, edible insects, entomophagy, food neophobia, willingness to eat

## Abstract

Insects have long been consumed as part of the diets of many Asian, African, and South American cultures. However, despite international agencies such as the Food and Agriculture Organization of the United Nations advocating the nutritional, environmental, and economic benefits of entomophagy, attitudinal barriers persist in Western societies. In Australia, the indigenous ‘bush tucker’ diet comprising witchetty grubs, honey ants, and Bogong moths is quite well known; however, in most Australian locales, the consumption of insects tends to occur only as a novelty. Therefore, this study aimed to investigate the awareness and acceptance of insects as food. An online survey of 820 consumers found that 68% of participants had heard of entomophagy, but only 21% had previously eaten insects; witchetty grubs, ants, grasshoppers, and crickets were the most commonly tasted insects. Taste, appearance, safety, and quality were identified as the factors that were most likely to influence consumer willingness to try eating insects, but consumer attitudes towards entomophagy were underpinned by both food neophobia (i.e., reluctance to eat new or novel foods) and prior consumption of insects. Neophobic consumers were far less accepting of entomophagy than neophilic consumers, while consumers who had previously eaten insects were most accepting of insects as food. Incorporating insects into familiar products (e.g., biscuits) or cooked meals also improved their appeal. Collectively, these findings can be used by the food industry to devise production and/or marketing strategies that overcome barriers to insect consumption in Australia.

## 1. Introduction

Approximately 1900 species of insect are harvested for consumption by an estimated two billion people, predominantly from developing countries in Asia, Africa, and South America [1,2]. Although their nutritional quality varies by species, developmental stage, and diet, insects tend to be high in protein, essential amino acids, fatty acids, vitamins, and minerals [1,3,4,5]. As a consequence, international agencies including the Food and Agriculture Organization of the United Nations are advocating for entomophagy (the consumption of insects) to meet the food demand associated with projected global population growth [1,6,7], since the global population is expected to reach nine billion by 2050 [6,7]. Compared with traditional animal-based protein sources (i.e., livestock, poultry, and fish), insects also afford significant environmental and economic benefits. Insect rearing produces substantially less greenhouse gas and ammonia emissions, and requires less water, feed, and space per kilogram of protein than conventional meat production [1]. However, despite these benefits, major attitudinal barriers to entomophagy persist in Western societies [1,3,8].

The traditional diet of indigenous Australians (colloquially termed the ‘bush tucker’ diet) comprises witchetty grubs, honey ants, and Bogong moths (*Endoxyla leucomochla, Myrmecocystus mexicanus,* and *Agrotis infusa*, respectively) [9], but the consumption of insects in Australia otherwise occurs only as a novelty. Perceptions of insects as pests [10], together with concerns that insects are dirty, disgusting, and unsafe [11,12], negatively influence the acceptance of insects as food. Consumer reluctance to eat insects has been attributed to underlying feelings of disgust [13,14,15,16], which are often associated with perceptions of danger, i.e., insects are considered to be dirty, unhygienic, and/or carriers of disease [1]. Thus, their consumption is thought to increase the risk of infection or contamination [14,16,17]. These attitudes are often deeply entrenched; improving the acceptance of unappealing foods is therefore a significant challenge [18]. The Food Neophobia Scale (FNS) is a psychometric instrument that is used to measure reluctance to eat, or avoidance of, new or novel foods [19]. Socio-demographic factors, such as sex, age, level of education, and ethnicity can influence food neophobia [20] in general, and acceptance of insects as food in particular [13,21,22]. In a study profiling consumers who were willing to adopt insects as a meat substitute, Verbeke identified younger males, with weak preferences for meat, low levels of neophobia, and concerns over the environmental impact of their food choices, as early adopters [23]. Familiarity with entomophagy also increases consumer readiness to adopt insects as food [23,24].

It is important to recognize that food preferences are not only motivated by psychological factors (e.g., cultural conditioning, food neophobia, and personal values/experiences); they are also motivated by factors related to the product, including price, appearance, taste/flavor, nutritional qualities, availability, and perceivable benefits [25]. The visual appearance and texture of food also plays an important role in determining consumer acceptance [17,26], with taste expectations found to be strong predictors of willingness to try edible insects [13]. Not surprisingly, the preparation and/or presentation of insects can also impact acceptance and/or liking, both positively and negatively [13,21,22,24]. 

Further research is needed to overcome the attitudinal barriers that are associated with eating insects in order to realize the nutritional, environmental, and economic benefits associated with entomophagy, and safeguard global food security. Given the prevalence of meat consumption in Western societies, there is significant potential for any uptake of entomophagy to achieve positive outcomes for the environment. The incorporation of insects into Western diets might also counter the decline in insect consumption that is observed in developing countries due to ‘Westernization’ [23]. This study therefore investigated: (i) Australian consumers’ awareness of entomophagy and (ii) the factors that are most likely to encourage acceptance of insects as food; these include not only demographic factors (including neophobia), but also factors that are related to the type and qualities of edible insects, together with the perceived importance of societal benefits. These insights can be used by the food industry to devise production and/or marketing strategies that enhance the consumer acceptance of insects and products containing insect-based ingredients, thereby fostering the uptake of entomophagy.

## 2. Materials and Methods

### 2.1. National Survey

An online survey was conducted to investigate Australian consumers’ awareness and acceptance of insects as food. Consumers (*n* = 820) were recruited nationally via a market research company (Valued Opinions, Forrest, ACT, Australia), with participants from: New South Wales (*n* = 251, 30.6%); the Northern Territory (*n* = 8, 1.0%); Queensland (*n* = 112, 13.7%); South Australia (*n* = 119, 14.5%); Tasmania (*n* = 20, 2.4%); Victoria (*n* = 215, 26.2%); and Western Australia (*n* = 95, 11.6%). Inclusion criteria required survey participants to be at least 18 years of age and residents of Australia. The survey was administered via SurveyMonkey (San Mateo, CA, USA) and comprised six sections. The first section captured consumer demographics via questions related to sex, age, education, household income, nationality, and protein consumption (Table 1). 

**Figure 1:**
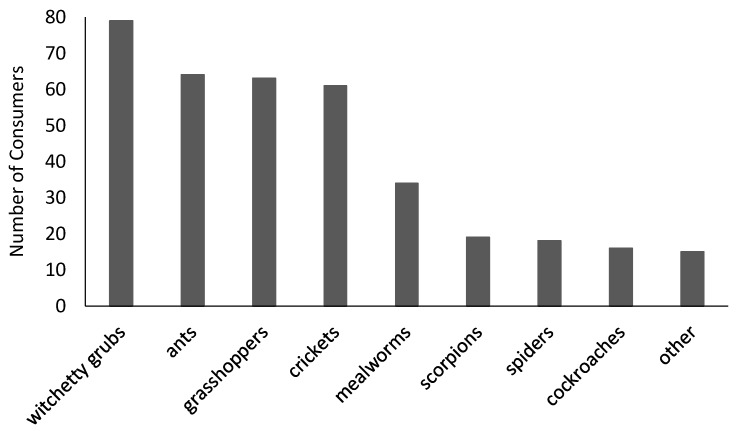
Histogram showing consumer (*n* = 169) consumption of edible insects.

The second section comprised the Food Neophobia Scale (FNS) developed by Pliner and Hobden [19]. The FNS comprises ten items: 1. I am constantly sampling new and different foods; 2. I don’t trust new foods; 3. If I don’t know what is in a food, I won’t try it; 4. I like foods from different countries; 5. Ethnic food looks too weird to eat; 6. At dinner parties, I will try a new food; 7. I am afraid to eat things I have never had before; 8. I am very particular about the foods I will eat; 9. I will eat almost anything; and 10. I like to try new ethnic restaurants. Consumers indicate their degree of agreement using seven-point Likert scales (where 1 = ‘strongly disagree’ and 7 = ‘strongly agree’). Total FNS scores (following the reversal of scores for items 1, 4, 6, 9, and 10) provide a measure of food neophobia, i.e., reluctance to eat new or novel foods [19]. Section three asked consumers whether they had previously heard of entomophagy or edible insects (Table 1), if they had previously eaten insects (Table 1), and if so, what they had eaten (Figure 1). In the last three sections of the survey, consumers responded to a series of statements (using seven-point category scales) to indicate: their willingness to try different edible insects (including scorpions and spiders, which are often designated as ‘edible insects’, despite their taxonomical classifications being Arachnida, not Insecta) or insect-based foods; their attitudes towards the benefits of incorporating insects into Western diets; and the extent to which a range of factors (adapted from those identified by Lensvelt and Steenbekkers [24]) might influence their willingness to try eating insects; with responses ranging from 1 (being ‘highly unlikely’ or ‘strongly disagree’) to 7 (being ‘highly likely’ or ‘strongly agree’). Consumers took approximately 15 min to complete the survey, and data were collected within a two-week period. Participation was voluntary; consumers received a small financial reward (<$10 AUD) in exchange for their involvement.

### 2.2. Data Analysis

Data were analyzed via a combination of descriptive techniques (means, frequencies, percentages) using Microsoft Excel, including segmentation of consumers on the basis of factors such as sex, age, ethnicity, prior consumption of insects, and food neophobia, as well as analysis of variance (ANOVA) using GenStat (15th Edition, VSN International Limited, Herts, UK).

## 3. Results and Discussion

### 3.1. Consumer Demographics

A slightly higher proportion of consumers were male (55%), but there was otherwise good representation across the different age, education, and income groups, with the exception of the lowest education level (primary school) and the highest income level (>$200,000), which is not unexpected (Table 1). The majority of consumers (79.4%) identified as Australians/New Zealanders, while Asian, English/Irish/Scottish, and European ethnicities comprised 10.5%, 10.4% and 7.6% of consumers, respectively. Only three consumers (0.4%) indicated they were indigenous (Aboriginal). A further 28 consumers nominated other ethnicities. Given the potential for edible insects to provide an alternative source of protein to meat/fish, consumers’ protein consumption was included as an additional demographic characteristic. With the exception of wild meat (kangaroo, deer, goat), which was eaten by 42% of consumers, responses indicated that each of the types of protein were consumed by more than 70% of consumers. Only 12 consumers (hereafter ‘non-meat eating consumers’) indicated they did not eat meat, fish, or seafood. However, given the self-selecting nature of recruitment, it is not surprising that a survey concerning edible insects did not attract many non-meat eating consumers (i.e., vegetarians or vegans).

### 3.2. Consumer Awareness and Consumption of Edible Insects

Despite the majority of consumers (i.e., 68%) indicating an awareness of entomophagy or edible insects, only 21% (*n* = 169) had previously eaten insects (Table 1). Witchetty grubs were the most commonly consumed edible insect, followed by grasshoppers, crickets, and ants (Figure 1). Mealworms, cockroaches, scorpions, and spiders had been eaten by considerably fewer consumers, which likely reflects the reduced availability and/or sensory appeal of these particular edible insects. Additional responses that were provided by a further 15 consumers (classified as ‘other’) comprised snails (*n* = 6), larvae, wasps, locusts, caterpillars, Bogong moths, and worms (*n* ≤ 2 each).

When the awareness and consumption of edible insects was considered according to the sex, age, ethnicity, and protein consumption of consumers (Table S1), several interesting trends were observed. The same proportion of male and female consumers had heard of entomophagy or edible insects, but a higher proportion of males had consumed edible insects than females, i.e., 27% compared with 13%. Indeed, of the 169 consumers who indicated that they had previously consumed insects, the majority (*n* = 120, 71%) were male (Table 1). This finding likely reflects a combination of males having more adventurous taste orientations and females being more disgusted by the idea of consuming insects, as suggested by Verbeke [23]. Interestingly, younger and older consumers, i.e., those aged <35 and ≥55 years respectively, had similar levels of both awareness and consumption (Table S1); however, a slightly higher proportion of the consumers who had previously eaten insects were aged ≥55 years (Table 1). Previous studies report conflicting results regarding the influence of age on consumer willingness to eat insects. Some studies suggest that the readiness to adopt insects as an alternative to meat is stronger in younger age groups [21,23]. In contrast, another study found that age did not significantly influence consumer acceptance of insects; instead, a strong cultural bias towards insects was identified [13]. In the current study, awareness of entomophagy was not strongly influenced by ethnicity, albeit English/Irish/Scottish consumers were slightly less aware than those from other ethnicities (Table S1). However, consumers from European backgrounds were less likely to have previously consumed edible insects, while consumers of Asian ethnicity were more likely to have eaten insects (15% and 30% respectively, compared with 21% for the total sample, Table S1). This finding suggests ethnicity influences consumers’ prior consumption of insects; which might relate to the availability of edible insects in certain cultural settings. Non-meat eating consumers were also less likely to have heard of entomophagy, but with such a small sample size, i.e., *n* = 12, this result is not conclusive. Again, given the self-selecting nature of recruitment, it is not surprising that a study concerning edible insects did not attract many non-meat eating consumers.

### 3.3. Segmentation of Consumers Based on Food Neophobia and Prior Consumption of Insects

Consumers were segmented according to their responses to questions from the FNS. Previous studies have taken different approaches to segmentation based on food neophobia. In some studies, consumers are designated as being neophobic or neophilic using mean or median FNS scores (e.g., [27,28]), whereas other studies applied cut-off scores (e.g., [29]). In the current study, consumers with FNS scores within the upper and lower quartiles were segmented as neophobic and neophilic consumers, respectively, to accentuate the variation inherent amongst the total sample. In the present study, 208 consumers (25.4% of the total sample) with FNS scores ≥40 were classified as food neophobes, and 214 consumers (26.1% of the total sample) with FNS scores of ≤25 were classified as food neophiles. Demographic data for neophobic and neophilic consumers were relatively similar (Table 1), albeit the neophilic consumer segment tended to be more educated, and comprised a higher proportion of consumers who identified as Asian or European, and fewer consumers who identified as English/Irish/Scottish, than the neophobic consumer segment. Not surprisingly, the protein consumption of neophobic and neophilic consumers also differed considerably. A higher proportion of neophiles consumed each of the protein types compared with neophobes; these proportions were substantially higher in the case of wild meat (64% versus 25%) and other seafood/shellfish (93% versus 46%).

Previous studies have discussed the influence of food neophobia on consumer acceptance of insects as food [13,22,23,30], and similar results were obtained in the current study (Table 1). A higher proportion of neophilic consumers (*n* = 169, 79%) indicated awareness of entomophagy than neophobic consumers (*n* = 114, 55%); meanwhile, neophiles were three times more likely to have consumed edible insects compared with neophobes. As such, subsequent analyses of consumer data compared responses from the neophobic and neophilic consumer segments. Since the previous consumption of insects has been shown to favorably influence attitudes towards entomophagy [24], an additional segment comprising consumers who indicated they had eaten insects (hereafter ‘insect eating consumers’) was also identified (Table 1). As indicated above, a much higher proportion of these consumers were male (71%). This segment also comprised higher proportions of older, more educated and Asian consumers. The average FNS score for insect eating consumers was 27, with 78 (46%) and 22 (13%) of these consumers being classified as neophilic and neophobic, respectively (data not shown), using the segmentation criteria based on FNS scores outlined above. 

### 3.4. Consumer Willingness to Try Edible Insects

Consumers were asked to indicate their willingness to try different types of edible insects. Flavored insects, chocolate-coated insects, biscuits made with insect flour, and a meal containing insects were included as options, to determine the extent to which, if any, insect appearance and/or presentation, e.g., whole insects versus the incorporation of insects in unrecognizable forms, might influence consumer attitudes. Significant differences were observed between the average scores given for the various edible insects and insect-based products (Table 2); from 2.08 for cockroaches to 3.90 for biscuits made with insect flour. It is perhaps not surprising that consumers were least willing to try cockroaches and spiders, given that both are generally considered to be pests, and in the case of cockroaches, indicators of poor food quality and/or spoilage. Certainly, health concerns have previously been identified as a perceived risk that is associated with eating insects [24]. The same study also demonstrated that consumers were more likely to eat insects when they were either mixed into a dish or unrecognizably incorporated into a product. Similar results were obtained in the current study, with consumers indicating they would be most willing to try a biscuit made with insect flour, followed by a cooked meal containing insects. Consumers were more willing to try a flavored insect than any of the whole insects that were suggested. This finding might indicate concerns related to taste, although chocolate-coating insects did not significantly increase their appeal; consumers rated their willingness to try chocolate-coated insects, crickets, ants, and witchetty grubs equally.

Whereas mean consumer responses were below the neutral score of four for each of the edible insects and insect-based products, individual consumer responses ranged from one to seven, i.e., spanned the seven-point category scale (data not shown). This highlights the inherent diversity in consumer attitudes, and thus the need for segmentation. Therefore, analysis of variance was performed on subsets of consumer data to compare the willingness of neophobic, neophilic, and insect eating consumers to try insects and insect-based products (Table 2). Mean responses ranged from 1.75 to 2.80 for neophobes, and from 2.55 to 4.88 for neophiles; scores for neophobic consumers were significantly lower than for neophilic consumers in all of the cases (*p* < 0.001). Responses from insect eating consumers ranged from 3.11 to 5.30, and in all of the cases were significantly more positive than for both neophobic and neophilic consumers (*p* ≤ 0.025). Irrespective of segmentation, again, consumers were least willing to try cockroaches and spiders, and were most willing to try biscuits made with insect flour and a cooked meal comprising insects. However, neophobic consumers were largely unwilling to try any of the edible insects or insect-based products, with few significant differences observed amongst their mean responses. In contrast, neophilic and insect eating consumers were more discerning. Mean responses for neophiles were at or above the neutral score of 4 (±0.1) for all of the insect-based products and most of the edible insects; only mealworms, scorpions, spiders, and cockroaches were considered unfavorably. Insect eating consumers only viewed cockroaches, spiders, and scorpions unfavorably; i.e., the edible insects that were most likely to elicit perceptions of risk and/or disgust.

The influence of demographic factors (sex, age, ethnicity, and protein consumption) on willingness to try edible insects and insect-based products were also considered (Table S2). Again, male consumers were more likely to try eating insects than female consumers, while similar responses were observed for younger and older consumers. However, older consumers were more willing to try witchetty grubs, biscuits made with insect flour, and a cooked meal made with insects. Some differences were observed amongst consumers of different ethnicities. Asian consumers were usually, but not always, more willing to try eating different types of insects than consumers from other cultural backgrounds; this finding is in agreement with a previous cross-cultural study comparing Chinese and German consumers’ attitudes towards entomophagy [13]. As expected, responses from non-meat eating consumers were consistently lower than for meat-eating consumers, ranging from 1.92 to 2.31 compared with 2.09 to 3.93, respectively.

### 3.5. Factors Influencing Consumer Willingness to Try Edible Insects

The nutritional, environmental, and economic benefits associated with entomophagy are well documented in both the scientific literature (e.g., [1,3,4,5,6]) and reports from international agencies such as the Food and Agriculture Organization of the United Nations [7,10]. However, it is not clear to what extent consumer awareness of the broader benefits of entomophagy might foster the acceptance of insects as food. It has been suggested that informing consumers of the benefits of eating insects increases their intention to try eating insects [15]. However, Deroy, Reade, and Spence argue that consumer food choices are driven by taste preferences and exposure, and so attitudinal barriers are unlikely to be overcome by educational strategies alone [14]. Thus, consumer attitudes towards the justifications made for eating insects, and the factors that were likely to influence willingness to try insects, were explored.

Collectively, consumers neither agreed nor disagreed with the reasons that were suggested for incorporating insects into Western diets, with mean responses ranging from 4.09 to 4.47 (Table 3). However, significant differences were observed between the attitudes of neophobic and neophilic consumers (*p* < 0.001). Neophobic consumers’ responses were consistently below the neutral score of 4 (3.51 to 3.81), whereas neophilic consumers’ responses were more favorable (4.47 to 5.09). Responses from insect eating consumers ranged from 4.99 to 5.41, and were again significantly higher than for the neophilic segment (*p* < 0.05).

In all of the cases, consumer agreement was highest for environmental sustainability, but there were few significant differences amongst responses given for each of the proposed benefits within consumer segments. Animal welfare was an exception to this trend, with significantly lower responses observed for both the total sample and the neophilic consumer segment for this benefit. These results suggest that either our consumers did not perceive any tangible benefits associated with the consumption of insects, or more likely, other factors were (negatively) influencing their attitudes towards the consumption of insects. Therefore, these results indicate that the broader benefits of entomophagy are not likely to be meaningful drivers of consumer acceptance. This finding is in agreement with the argument put forward by Deroy, Reade, and Spence, that “stressing the sustainability and nutritional value of insects as a source of food is unlikely to provide sufficient motivation to drive through a change in diet” [14].

Consumers were asked to rate to what extent a range of factors might influence their willingness to try eating insects (Table 4). Of the various factors offered, insect taste/flavor was considered the most influential, followed by food safety, insect appearance, quality, and nutritional value. Mean responses for these factors were at or above the neutral score of 4 (±0.1), whereas other factors were considered less influential, with scores between 3.60 and 3.82. As before, significant differences were observed between responses given by neophobic and neophilic consumers (*p* < 0.001), and by neophilic and insect eating consumers (*p* ≤ 0.015). Mean responses from neophobic consumers ranged from 2.86 (for price) to 3.30 (for taste/flavor), but were not significantly different; i.e., all of the factors were considered equally unfavorably. This finding suggests that attempts to persuade these consumers to consider eating insects would likely be futile; their negative attitudes and/or phobias towards entomophagy are simply too entrenched. In contrast, mean responses from neophilic and insect eating consumers ranged from 4.21 to 5.06 and 4.73 to 5.49, respectively. Therefore, consumers within these segments represent a far more viable target market, particularly those consumers who have previously eaten insects.

It is not surprising that both the appearance and anticipated taste of insects were found to influence the acceptance of insects as food. This finding is not only in agreement with numerous previous studies [2,14,17,21,22,24,30], it was also consistent with ratings obtained for willingness to try different edible insects and insect-based products, which indicated a preference for biscuits made from insect flour and cooked meals containing insects (Table 2). Consumers not only perceive the inclusion of insects in recognizable products as being more appealing than whole insects, their familiarity with the preparation methods used to make these types of products (e.g., baking) and/or ability to modify taste/flavor through the addition of other ingredients likely helps to overcome concerns associated with taste/flavor, food safety, and quality when consuming insects. Lensvelt and Steenbekkers evaluated the importance of various factors in determining food purchasing and consumption decisions, including convenience, i.e., the ease with which consumers can source, store, and prepare insects for consumption [24]. The provision of ready-made foods such as biscuits, bread, or pasta derived from insect-based flour might also facilitate the uptake of entomophagy by making insects more accessible in terms of availability, storage, and preparation. Consideration will need to be given to the labeling of foods made from insects. The presence of insects in foods should be explicit, so as to inform consumers with food allergies (the chitin present in the exoskeleton of edible insects can elicit allergic reactions similar to those from shellfish [1]). However, the visual and descriptive cues on packaging may negatively impact consumer perceptions of risk, and therefore purchase intent [14,17]; at the same time, ambiguous references to insect ingredients are deceptive [17].

That environmental benefits were amongst the least influential factors suggests that consumers are not willing to forgo palatability for the sake of the environment. This finding is consistent with other research exploring “ethical consumerism”, which suggests that the sensory aspects of food maintain importance, even when consumers are highly motivated about an issue, such as animal welfare for example [31]. Previous studies have highlighted tensions between the ideas of the consumer and the citizen [32,33], suggesting that even with efforts to promote the broader benefits of entomophagy and education to change the mindset of those who perceive edible insects as a novelty, the acceptance of insects as food is unlikely, unless sensory (taste/flavor), health, and quality-related concerns are also addressed. Indeed, convincing consumers that insects can be pleasurable to eat may yet prove to be the most effective strategy in promoting the adoption of entomophagy [14].

## 4. Conclusions

The results from this study demonstrate there is potential for edible insects to be introduced into Australian diets, with a considerable proportion of the population expected to exhibit neutral or positive attitudes towards entomophagy. While consumers are not likely to try insects that they perceive to be pests and/or harmful, e.g., cockroaches, spiders, or scorpions, insects such as crickets, ants, and witchetty grubs would be considered more favorably. Consumers would also be more accepting of insects as food if they were incorporated into familiar products, e.g., biscuits, bread, or pasta made from insect-based flour, or as part of cooked meals. These options overcome consumer concern around the appearance, taste, and safety of edible insects, thereby increasing their appeal. These findings can be used by industry to inform production strategies; i.e., which insects can be incorporated into which foods, so as to enhance consumer appeal. Although only 21% of the consumers surveyed in the current study had previously eaten insects, this segment was the most accepting of insects as food. Thus, providing opportunities for consumers to try insects or insect-based products is likely to be the most effective marketing strategy to foster the acceptance of insects as food, at least in the short term.

## Figures and Tables

**Table 1:** Consumer demographics, awareness, and consumption of edible insects.

	Total Sample *n* = 820	Neophobic Consumers FNS ≥40, *n* = 208	Neophilic Consumers FNS ≤25, *n* = 214	Insect Eating Consumers ^1^ *n* = 169
*Sex*				
female	45	46	45	29
male	55	54	55	71
*Age (years)*				
18–24	11	14	10	11
25–34	20	19	22	18
35–44	25	27	24	27
45–54	19	16	21	17
55–64	15	14	12	12
≥65	10	9	11	15
*Education*				
primary school	1	2	2	1
secondary school	24	27	20	17
technical/trade certificate	31	34	29	22
undergraduate university	31	27	34	40
postgraduate university	13	10	15	20
*Household income (AUD)* ^2^				
≤50,000	25	31	21	21
50,001–100,000	35	32	27	33
100,001–150,000	19	13	21	22
150,001–200,000	7	7	7	9
>200,000	3	2	4	3
*Ethnicity* ^3^				
Australian/New Zealander	79.4	80.3	79.4	78.1
Indigenous (Aboriginal)	0.4	0.5	0.0	0.0
English/Irish/Scottish	10.4	13.5	10.7	9.5
European	7.6	7.7	8.9	5.3
Asian	10.5	7.7	11.2	15.4
other	3.4	3.8	4.2	0.5
*Protein consumption*				
red meat (beef, lamb)	93	88	99	92
white meat (chicken, turkey)	95	91	100	93
white meat (pork)	80	68	92	84
wild meat (kangaroo, deer, goat)	42	25	64	65
fish	89	83	99	91
other seafood (shellfish)	71	46	93	81
none of the above	1.5 (*n* = 12)	1.0 (*n* = 2)	0.0 (*n* = 0)	1.2 (*n* = 2)
*Have you previously heard of entomophagy or edible insects?*				
yes	68	55	79	89
no	32	45	21	11
*Have you previously consumed edible insects?*				
yes	21	11	36	100
no	79	89	64	0

Data are presented as percentages. ^1^ Consumers who indicated they had previously consumed insects. ^2^ 11% of consumers (*n* = 93) elected not to disclose their household income. ^3^ Consumers could nominate up to two ethnicities.

**Table 2:** Consumer willingness to try edible insects and insect-based products.

	Total Sample *n* = 820	Neophobic Consumers *n* = 208	Neophilic Consumers *n* = 214	Insect Eating Consumers ^1^ *n* = 169	*p* ^2^
*If you had the opportunity, how likely would you be to try a...*					
mealworm	2.68 e	1.95 b	3.51 e	4.16 d	0.001
cricket	3.00 d	1.98 b	4.00 cd	4.64 c	0.002
ant	3.01 d	1.99 b	3.96 cd	4.66 bc	<0.001
cockroach	2.08 g	1.75 b	2.55 f	3.11 e	0.004
witchetty grub	2.98 d	2.09 b	3.92 d	4.63 c	<0.001
scorpion	2.47 f	1.88 b	3.16 e	3.81 d	0.002
spider	2.17 g	1.76 b	2.77 f	3.40 e	0.002
flavored insect	3.28 c	2.15 b	4.33 bc	4.92 abc	0.005
chocolate-coated insect	3.03 d	2.10 b	3.95 cd	4.60 c	0.003
biscuit made with insect flour	3.90 a	2.80 a	4.88 a	5.30 a	0.025
cooked meal made with insects	3.56 b	2.42 a	4.57 ab	5.04 ab	0.011

Values are means, where 1 = highly unlikely and 7 = highly likely. Different letters within a column indicate a statistically significant difference (*p* = 0.05, one-way ANOVA). ^1^ Consumers who indicated they had previously consumed insects. ^2^ *p* values shown are for ANOVA for responses from neophilic vs. insect eating consumers; *p* values for ANOVA for responses from neophobic vs. neophilic consumers were always <0.001.

**Table 3:** Consumer attitudes towards the benefits of eating insects.

	Total Sample *n* = 820	Neophobic Consumers *n* = 208	Neophilic Consumers *n* = 214	Insect Eating Consumers ^1^ *n* = 169	*p* ^2^
*Insects should be incorporated into Western diets to address issues related to...*					
nutrition	4.33 ab	3.51	4.94 a	5.40	0.004
food security	4.16 bc	3.53	4.76 ab	5.21	0.009
environmental sustainability	4.47 a	3.81	5.09 a	5.41	0.047
reduced food wastage	4.35 a	3.74	4.87 a	5.34	0.008
scarcity of agricultural land	4.35 a	3.75	4.90 a	5.31	0.015
animal welfare	4.09 c	3.56	4.47 b	4.99	0.004

Values are means, where 1 = strongly disagree and 7 = strongly agree. Different letters within a column indicate a statistically significant difference (*p* = 0.05, one-way ANOVA). ^1^ Consumers who indicated they had previously consumed insects. ^2^ *p* values shown are for ANOVA for responses from neophilic vs. insect eating consumers; *p* values for ANOVA for responses from neophobic vs. neophilic consumers were always <0.001.

**Table 4:** Factors influencing consumer willingness to try eating insects.

	Total Sample *n* = 820	Neophobic Consumers *n* = 208	Neophilic Consumers *n* = 214	Insect Eating Consumers ^1^ *n* = 169	*p* ^2^
*To what extent would the following factors influence your willingness to try eating insects?*					
price	3.60 e	2.86	4.26 ef	4.81 cd	0.002
quality	3.98 b	3.00	4.79 abc	5.23 ab	0.013
nutritional value	3.92 bc	2.97	4.62 bcde	5.08 bc	0.011
food safety	4.07 b	3.11	4.75 abcd	5.22 ab	0.009
taste/flavor	4.29 a	3.30	5.06 a	5.49 a	0.014
environmental benefits	3.66 de	2.90	4.21 f	4.73 d	0.004
product approval	3.82 cd	2.94	4.37 ef	4.99 bcd	<0.001
absence of additives	3.78 cde	2.94	4.41 def	4.99 bcd	0.002
availability	3.76 cde	2.89	4.45 cdef	4.98 bcd	0.003
appearance	4.09 b	3.20	4.84 ab	5.27 ab	0.012

Values are means, where 1 = highly unlikely and 7 = highly likely. Different letters within a column indicate a statistically significant difference (*p* = 0.05, one-way ANOVA). ^1^ Consumers who indicated they had previously consumed insects. ^2^ *p* values shown are for ANOVA for responses from neophilic vs. insect eating consumers; *p* values for ANOVA for responses from neophobic vs. neophilic consumers were always <0.001.

## Supplementary Materials

The following are available online at http://www.mdpi.com/2075-4450/9/2/44/s1, Table S1: Awareness and consumption of edible insects by consumers segmented according to their sex, age, ethnicity and protein (meat) consumption. Table S2: Willingness of different consumer segments to try eating insects and insect-based products.
