# Supplementary material for: Australian Consumers’ Awareness and Acceptance of Insects as Food
Source: Insects. 2018 Apr 19;9(2):44. doi: 10.3390/insects9020044 (PMC6023301; doi:10.3390/insects9020044)
Supplement: Supplementary file 1 [file insects-09-00044-s001.pdf]

insects

MDPI

Table S1. Awareness and consumption of edible insects by consumers segmented according to their sex, age, ethnicity and protein (meat) consumption.

	female consumers n = 368	male consumers n = 452	younger consumers (<35 years) n = 250	older consumers (≥55 years) n = 205	Australian/ New Zealand consumers n = 651	English/Irish/ Scottish consumers n = 85	European consumers n = 62	Asian consumers n = 86	meat-eating consumers n = 808	non-meat eating consumers[1] n = 12
Have you previously heard of entomophagy or edible insects?										
yes	68	68	69	67	70	62	68	70	68	58
no	32	32	31	33	30	38	32	30	32	42
Have you previously consumed edible insects?										
yes	13	27	20	22	20	19	15	30	21	17
no	87	73	80	78	80	81	85	70	79	83

Data are presented as percentages. [1] Consumers who indicated they did not consume animal-based proteins.

Table S2. Willingness of different consumer segments to try eating insects and insect-based products.

	female consumers n = 368	male consumers n = 452	younger consumers (<35 years) n = 250	older consumers (≥55 years) n = 205	Australian/ New Zealand consumers n = 651	English/Irish/ Scottish consumers n = 85	European consumers n = 62	Asian consumers n = 86	meat-eating consumers n = 808	non-meat eating consumers[1] n = 12
If you had the opportunity, how likely would you be to try a...										
mealworm	2.40	2.93	2.57	2.55	2.73	2.57	2.35	2.62	2.70	2.15
cricket	2.64	3.30	3.03	3.18	3.00	2.76	2.65	3.24	3.01	2.31
ant	2.66	3.29	3.03	3.21	3.02	2.70	2.68	3.34	3.02	2.31
cockroach	1.80	2.32	2.21	2.10	2.08	1.95	1.90	2.25	2.09	1.92
witchetty grub	2.50	3.37	2.70	3.34	3.04	2.80	2.59	2.67	2.99	2.08
scorpion	2.02	2.83	2.57	2.54	2.42	2.30	2.37	2.88	2.47	2.00
spider	1.76	2.51	2.17	2.26	2.14	2.01	1.97	2.43	2.18	1.92
flavored insect	2.92	3.58	3.24	3.52	3.31	3.12	2.83	3.36	3.30	2.00
chocolate-coated insect	2.75	3.25	3.04	3.21	3.06	2.83	2.57	2.91	3.04	2.00
biscuit made with insect flour	3.68	4.09	3.76	4.21	3.94	3.96	3.37	3.76	3.93	2.31
cooked meal made with insects	3.26	3.81	3.45	3.82	3.58	3.58	3.16	3.60	3.58	2.15

Data are presented as percentages. [1] Consumers who indicated they did not consume animal-based proteins.

© 2018 by the authors. Submitted for possible open access publication under the terms and conditions of the Creative Commons Attribution (CC-BY) license (http://creativecommons.org/licenses/by/4.0/).
